# Autologous stromal vascular fraction therapy for rheumatoid arthritis: rationale and clinical safety

**DOI:** 10.1186/1755-7682-5-5

**Published:** 2012-02-08

**Authors:** Jorge Paz Rodriguez, Michael P Murphy, Soonjun Hong, Marialaura Madrigal, Keith L March, Boris Minev, Robert J Harman, Chien-Shing Chen, Ruben Berrocal Timmons, Annette M Marleau, Neil H Riordan

**Affiliations:** 1 Medistem Panama, Panama City, Panama; 2 Indiana University, Indiana, USA; 3 University of San Diego, San Diego, CA, USA; 4 Vet-Stem Inc, Poway, CA, USA; 5 Division of Hematology and Oncology, Loma Linda University, School of Medicine, Loma Linda, CA, USA; 6 Senacyt, Panama City, Panama, USA; 7 Institute for Molecular Medicine, Huntington Beach, CA, USA

## Abstract

Advancements in rheumatoid arthritis (RA) treatment protocols and introduction of targeted biological therapies have markedly improved patient outcomes, despite this, up to 50% of patients still fail to achieve a significant clinical response. In veterinary medicine, stem cell therapy in the form of autologous stromal vascular fraction (SVF) is an accepted therapeutic modality for degenerative conditions with 80% improvement and no serious treatment associated adverse events reported. Clinical translation of SVF therapy relies on confirmation of veterinary findings in targeted patient populations. Here we describe the rationale and preclinical data supporting the use of autologous SVF in treatment of RA, as well as provide 1, 3, 6, and 13 month safety outcomes in 13 RA patients treated with this approach.

## Introduction

Increasing number of reports support the possibility of utilizing adult stem cell therapy not only for treatment of degenerative conditions, but also as a means of addressing underlying inflammation or autoimmune conditions [1-8]. Unfortunately, stem cell therapy is often complicated by the need for complex laboratories, processing procedures and clean rooms. The potential drawbacks of allogeneic donor approaches include the possibility of eventual rejection of the cellular graft [9-12], as well as limitation of efficacy due to trophic effects but not de novo tissue generation [13-15]. Conversely, adult stem cell based approaches, particularly using bone marrow, are limited to the relatively small number of progenitor cells within the bone marrow. While bone marrow mononuclear cell administration appears to be effective in conditions where cells are locally implanted, such as intramyocardial [16-18], or intramuscular in critical limb ischemia [19-21], the intravenous administration of non-expanded bone marrow has not been performed with efficacy in systemic conditions without prior myeloablation of the recipient. One way of circumventing this problem is to expand autologous stem cells prior to implantation. Unfortunately, besides issues of cost and practicality, there is a risk that the in vitro manipulation could be linked to contamination, as well as genomic alterations of the cells, leading to transformation.

Several studies have used bone marrow derived mesenchymal stem cells (MSC) for various conditions including type 2 diabetes [22], osteoarthritis [23], stroke [24], and amyotrophic lateral sclerosis [25]. This procedure requires expansion of the MSC compartment in vitro and therefore adds an element of complexity to the treatment. A much simpler procedure, for which adipose tissue is uniquely suited, is the administration of autologous, non-expanded cellular fraction. The rationale behind this derives from observations that: a) adipose tissue contains substantially higher numbers of MSC compared to bone marrow [26]; b) MSC from adipose tissue do not appear to decrease in number as a result of age [27,28]; and c) adipose tissue contains unique populations of cells including high concentrations of endothelial progenitor cells and T regulatory (Treg) cells that express up to 100-fold higher levels of the immune suppressive cytokine IL-10 as compared to circulating Tregs [29].

The adipose stromal vascular fraction (SVF) is comprised of the mononuclear cells derived from adipose tissue. This term is more than 4 decades old, used to describe the mitotically active source of adipocyte precursors [30,31]. SVF as a source of stem cells was first described by Zuk et al. who identified MSC-like cells in SVF that could be induced to differentiate into adipogenic, chondrogenic, myogenic, and osteogenic lineages [32]. Subsequent to the initial description, the same group reported after in vitro expansion, the SVF derived cells had surface marker expression similar to bone marrow derived MSC, comprising CD29, CD44, CD71, CD90, CD105/SH2, andSH3 and lacking CD31, CD34, and CD45 expression [33,34].

Reported clinical trials on adipose derived cells, to date, have all utilized ex vivo expanded cells, which share properties with bone marrow derived MSC [35-40]. MSC expanded from adipose tissue are equivalent, if not superior to bone marrow in terms of differentiation ability [41,42], angiogenesis stimulating potential [43,44], and immune modulatory effects [45]. Given the requirements and potential contaminations associated with ex vivo cellular expansion, a simpler procedure would be the use of primary adipose tissue derived cells for therapy. Indeed, it is reported that over 4000 horses and 4000 dogs with various cartilage and bone injuries have been successfully treated with autologous SVF [46]. In double blind studies of canine osteoarthritis statistically significant improvements in lameness, range of motion, and overall quality of life have been described [47,48].

If such approaches could be translated clinically, an easy-to-use autologous stem cell therapy could be implemented that is applicable to a multitude of indications. Indeed, this is the desire of commercial entities that are developing bench-top closed systems for autologous adipose cell therapy [49,50], which are presently entering clinical trials. Unfortunately, since the majority of scientific studies have focused on in vitro expanded adipose derived cells, relatively little is known about the potential clinical effects of the whole lipoaspirate that contains numerous cell populations besides MSC. From a safety perspective the process of autologous fat grafting has been commonly used in cosmetic surgery [51,52]. Therefore, administration of autologous heterogeneous adipose cellular fractions, which contain numerous cellular populations besides MSC, should be relatively innocuous. However, from an efficacy or disease-impact perspective, it is important to consider the various cellular components of adipose tissue and to develop a theoretical framework for evaluating activities that these components may mediate when administered systemically. For example, while attention is focused on the MSC component of adipose tissue, the high concentrations of monocytes/macrophages, and potential impact these may have on a clinical indication is often ignored [29].

In the published literature, the clinical use of systemically administered SVF cells has been reported in two pilot studies by our group. The first was a description of 3 patients suffering from multiple sclerosis who received intravenous administration of autologous adipose SVF as part of a cellular cocktail. All 3 patients reported significant improvement neurologically, and demonstrated an excellent safety profile [53]. Additionally, a 1 patient case report described a remission of RA subsequent to administration of autologous SVF as a monotherapy [29]. Here we will provide a description of adipose SVF components, provide a rationale for use, and describe safety at 1, 3, 6, and 12 months in a 13 patient retrospective analysis.

## The MSC component of adipose tissue

MSC are conventionally extracted from bone marrow sources as a cellular therapy for inflammatory associated conditions. Specifically, the most advanced clinical trials in the area of regenerative medicine have been performed by the company Osiris, whose main product is a "universal donor" MSC, termed "Prochymal"[54]. This cellular product has entered Phase III trials in graft versus host disease, and is currently being tested for heart failure [55]. Other bone marrow derived MSC-like products are in clinical trials, for example, Mesoblast is in Phase III assessing its Mesenchymal Precursor Cell for efficacy in post hematopoietic transplant graft failure, as well as in Phase II for heart failure [56]. Therapeutic advantages of MSC include their ability to migrate to injured tissue, in part via detections of hypoxia through the CXCR4-SDF-1 axis [57,58], differentiation activity into multiple tissues [59,60], release of trophic factors [61], inhibition of apoptosis [62-64], stimulation of angiogenesis [65], inhibition of inflammation [66], and stimulation of Treg activity [67]. Despite the advantages of the current approaches, bone marrow contains relatively small numbers of MSC, thus, as previously mentioned, therapeutics with bone marrow for systemic applications requires ex vivo expansion. Specifically, the bone marrow contains approximately 1/10,000 to 1/100,000 MSC per nucleated cells [68], whereas adipose tissue contains approximately 100-1000 fold higher MSC concentration, or approximately 50-100,000 MSC per ml [69]. Given the relative ease of extracting 500 ml of lipoaspirate, it is conceptually feasible to generate a 25-50 million cell dose of MSC, which is close to the systemic doses of MSC that are typically used in clinical trials of allogeneic expanded cells (eg. 50-100 million cells in various clinical trials) [34]. Conceptually, given that the MSC present in the SVF are autologous, one could envision higher therapeutic potential due to the lack of allo-immune clearance as compared to allogeneic MSC, although this needs to be assessed experimentally.

Adipose MSC contain several similarities and differences as compared to bone marrow derived MSC, although this area is still considered to be controversial. Specifically, in animal cardiac infarct models it has been demonstrated that that expanded adipose MSC are superior to bone marrow MSC in terms of stimulating angiogenesis, decreasing cardiac pathology, and stimulating VEGF and FGF secretion [70]. Using an in vivo lentiviral-labeled system, it was demonstrated that adipose derived MSC have a superior ability to BM derived MSC to integrate into cardiac muscle after injury, as well as to restore function [71]. In addition to specific propensities for differentiation, adipose tissue-derived MSC appear to be superior to bone marrow in terms of proliferative potential without loss of telomere length. Vidal et al. demonstrated that adipose MSC could multiply for almost twice as many cell passages without undergoing senescence as compared to bone marrow MSC [72].

Conversely, several authors have reported similarities between bone marrow and SVF MSC sources. For example, subsequent to exposure to chemotactic agents, both sources were reported to yield MSC possessing similar rates of migration [73]. The same study also demonstrated comparable ability to generate cartilage when treated under differentiation conditions. Another study reported exposure of bone marrow or adipose derived MSC to ischemia leads to the release of similar levels of angiogenic factors, as well as resistance to apoptosis when cultured in hypoxic environments [74]. Comparison of immunological properties led to the conclusion that when expanded, both BM and adipose derived MSC appear to have similar properties in terms of suppressing mixed lymphocyte reactions, inhibiting release of type 1 and inflammatory cytokines, as well as generating progeny cells that appear to be relatively immune privileged [75]. These data were confirmed by the group of Zhang et al. who compared cord blood, bone marrow, and adipose MSC and found almost identical ability to inhibit immune response assays in vitro [76]. In contrast, Najar et al. reported that Wharton Jelly and adipose derived MSC were superior immune suppressors as compared to BM MSC in terms of inhibiting lymphocyte proliferation and type 1 cytokine production [77,78]. An important consideration is that there is a great deal of variability between studies, not only in the tissue sources from which MSC are derived but also in terms of cell isolation, culture and expansion methods as well as donor-specific characteristics that could conceivably influence the activities and differentiation potential of these cells [79]. Therefore, one potential disadvantage of utilizing ex vivo-manipulated MSC is the potential for introducing more heterogeneity in their regenerative capabilities. For treatments involving autologous MSC, patient-to-patient differences in MSC function could also lead to variability in the clinical efficacy of treatments.

Clinically, adipose derived MSC have been used in treatment of 8 spinal cord injury patients in Korea where administration of autologous expanded MSC at doses of 400 million per patient did not elicit treatment associated adverse events during a 3-month follow-up [80]. Additionally, this study also reported genetic stability of MSC in vitro and lack of toxicity or tumorigenicity of MSC in immune deficient mice. Trivedi's group treated 11 patients with type 1 diabetes using a combination of autologous adipose derived MSC that were cultured in a pro-pancreatic differentiation media together with cultured bone marrow MSC. No adverse effects were noted over an average of 23 months follow-up period and a decrease in insulin requirements was noted [81]. Garcia-Olmo et al. reported a study where autologous expanded adipose MSC were administered to patients with complex perianal fistulas, with 35 of cryptoglandular origin and 14 associated with Crohn's disease. They observed that fistula healing occurred in 17 (71 percent) of 24 patients who received ASCs in addition to fibrin glue compared with 4 (16 percent) of 25 patients who received fibrin glue alone (relative risk for healing, 4.43; confidence interval, 1.74-11.27; *P *< 0.001). The proportion of patients with documented fistula healing was similar in Crohn's and non-Crohn's subgroups. ASCs were also more effective than fibrin glue alone in patients with a suprasphincteric fistulous tract (*P *= 0.001). Furthermore, quality of life scores were higher in patients who received ASCs than in those who received fibrin glue alone [82]. Due to the anti-inflammatory effects of MSC in general [83-86], and specifically the ability of MSC to inhibit graft versus host disease (GVHD) [87,88], Fang et al. reported a series of pilot cases in which patients with steroid refractory GVHD was successfully treated by administration of autologous adipose derived expanded MSC [89,90].

Thus it appears that the MSC component of adipose tissue possesses numerous preclinical and clinical therapeutic properties and may be an important component of the SVF cell population that is responsible for therapeutic effects observed after administration.

## Adipose tissue resident T regulatory (treg) cells

Treg cells are conventionally described as CD4+ cells possessing the transcription factor FoxP3 and capable of suppressing T cell activation, dendritic cell maturation, neutrophil activation, and antibody production. The fundamental role of Treg in controlling immunity can be illustrated by the fact that genetic mutations associated with loss of Treg function, such as FoxP3 mutations, are associated with autoimmunity in mouse and man [91-93]. Additionally, conditional ablation of the Treg compartment in genetically-engineered mice results in systemic organ autoimmunity [94]. Numerous autoimmune conditions enter remission as a result of increased Treg number and/or activity, whereas relapse is associated with reduction in number and/or activity. Specifically, this has been demonstrated in multiple sclerosis [95-99], rheumatoid arthritis [100-104], and lupus [105-107]. Given the importance of Treg cells in the control of autoimmunity, it would be useful to possess sources of Treg cells that are easily accessible and can be reintroduced into the patient for immune modulation. It has been previously demonstrated that high numbers of Treg cells are found in adipose tissue at concentrations much higher than other peripheral compartments such as blood or spleen [108]. Interestingly, adipose derived Treg contain approximately 100 fold higher concentrations of the immune regulatory effector cytokine IL-10 [109,110]. It is known that the adipose derived cytokines leptin and TNF-alpha inhibit Treg proliferation and activity in vivo [111,112]. The local effects of these cytokines would conceptually, be negated by liberating Treg from fat tissue followed by systemic re-administration, resulting in enhanced Treg activity. Administration of a large number of Treg cells with augmented in vivo proliferative and functional potential may result in a reduction of the threshold needed to attain tolerance to an ongoing immune response. For example, anti-CD3 antibodies have been reported to induce antigen-specific tolerance, despite the fact that the surge in Treg numbers was not antigen-specific [113]. Thus one conceptual advantage of utilizing SVF therapy would be not only the MSC content, which possesses various regenerative properties, but also Treg, which would enhance anti-inflammatory/tolerance inducing properties. Given that both MSC and Treg are considered to be tolerance-promoting, it may be feasible to consider that synergize of tolerance induction may be occurring when the two cell populations are co-administered in the form of SVF.

## Endothelial progenitor cell (EPC)

Aging and/or damaged blood vessel endothelium is constantly renewed by circulating cells termed endothelial progenitor cells (EPC). This notion gathered significant scientific following subsequent to a paper by Asahara et al. who demonstrated that BM-originating cells expressing VEGFR-2 and CD34 are capable of incorporating into sites of angiogenesis induced by wire injury or ischemia [114]. Therapeutic properties of EPC have been demonstrated in that administration of exogenous EPC increases vascular repair. This has been shown using in vitro generated EPC, or bone marrow as a source of EPC in myocardial infarct [115,116], stroke [117], lung injury [118-120], liver failure [121-123], and endothelial injury atherosclerotic models [124,125]. Furthermore, administration of growth factors that stimulate mobilization of bone marrow stem cells and EPC have demonstrated therapeutic benefit in animal models of ischemic disease [126,127] as well as endothelial damage [128]. Clinical trials using EPC or bone marrow as a source of EPC for cardiovascular conditions [129-132], have demonstrated some therapeutic benefit, although work is ongoing.

Historically, the bone marrow has been used as a source of EPC, however, numerous recent studies have demonstrated a high content of EPC in adipose tissue [133,134]. Functional demonstration of adipose EPC was performed in experiments in which CD34 expressing cells were sorted for from SVF. This cellular fraction was demonstrated to induce angiogenesis in immune compromised mice that were subjected to hindlimb ischemia. Mechanistically, the cells were identified as EPC based on ability to form endothelial colonies when cultured in vitro [135]. Numerous groups have reported that SVF contains cellular activity that stimulates angiogenesis, for example, Sumi et al. showed that administration of SVF but not adipocytes led to revascularization in the hindlimb ischemia model [136]. Other studies have shown that not only are EPC-like activities found in SVF [137], but also that conditioned media from SVF is capable of stimulating host angiogenesis [138,139]. It is reported that EPC in the SVF stimulate angiogenesis directly through differentiating into endothelial cells or through release of growth factors such as IGF-1, HGF-1 and VEGF [136,137,139,140]. Although back-to-back comparisons of bone marrow and adipose derived EPC for assessing angiogenic potential have not been performed, the substantially higher concentration of these cells in SVF supports the investigation of this tissue as a practical cell source for clinical applications.

## Rationale for clinical applications

Given that SVF represents a multi-cellular population containing MSC, Treg, and EPC, the potential for therapeutic utilization would include many conditions that require regeneration, immune modulation, and possibly angiogenesis.

We previously reported remission in a patient with rheumatoid arthritis who was treated with autologous SVF [29]. Animal studies using the collagen II model of RA have demonstrated that administration of MSC is associated with immune modulation [141-143], disease remission [144,145], and regeneration of cartilage [5,146]. Additionally, our group and others have reported that Treg cells are associated with induction of disease remission [100-102,104,147-151].

## Safety data

The study was a retrospective analysis of patients treated under the practice of medicine under doctor patient privilege. The protocols were approved by local and institutional committees and all patients signed informed consent forms explaining the unproven and experimental nature of the treatment. Retrospective chart analysis of the patients was approved by PEARL IRB (Indianapolis, Indiana).

Patients received the indicated amount of cells by intravenous injection (2x10^6 ^cells per ml diluted in Saline solution), intra-articular injection (2.5x10^6 ^cells per ml in each injured joint, diluted in Saline solution and the patient's own serum). Multiple injections of cells were given to increase the therapeutic efficacy. Follow-ups were performed for all patients at 1, 3, 6 and 12 months.

SVF cells were isolated and prepared under the guidelines of Good Tissue Practices 21 CFR 1271 as relates to sample screening and processing in the sterile flow hood, inside of a class 10000 clean room. SVF cells were isolated by first washing 500 cc of lipoaspirate with PBS and subsequently, the cells were transferred to 175 ml sterile centrifuge containers followed by the addition of collagenase solution for a final concentration of 0.048%. The centrifuge containers were sealed and placed in an elliptical shaker and incubated at 37 C for 60-80 minutes. The content of the tubes was filtered through a cell strainer into sterile 50 ml centrifuge tubes and centrifuged for 12 min at 800 rcf. During centrifugation, SVF cells formed a pellet in the bottom of the container while the adipocyte layer and debris remained suspended. Following centrifugation, the stromal cells were resuspended in 5 mL of autologous serum for enzyme inactivation then washed 2 times with PBS. The fraction used for intraarticular injection was incubated with buffer to lyse red blood cells and washed once more. All the cells were aliquoted in cryovials, frozen in liquid nitrogen and stored until use. Cells were assessed for viability, endotoxin, and contamination before treatment was performed. The patient was allowed to heal from the liposuction for one week. For each treatment session, after thawing the cells were rinsed with PBS and Human AB serum, diluted in saline solution and autologous serum, loaded into sterile syringes, and then transported in a controlled temperature cooler accompanied by the corresponding certificate and delivered to the physician for infusion.

Thirteen patients with rheumatoid arthritis were treated with 38-148 million SVF cells intravenously and intra-articularly (Table 1). Although no hematopoietic or biological abnormalities were noted, one of the patients reported facial flushing, fever and myalgia after a third of four injections. These symptoms all resolved spontaneously.

**Table 1:** Patient Treatments and Safety Outcome

Date of Birth	Date Treated	Total SVF Dose (x 10^6^) & route of injection *	Side Effects
		(SVF Dose × no. injections)	
6/24/1975	3/2/2010	128 IV (32 × 4)	Facial flushing, fever, myalgia (resolved after 3^rd ^injection)

1/15/1950	9/27/2010	120 IV (40 × 3)	None

4/10/1954	5/26/2010	90 IV (30 × 3)	None

7/4/1967	7/22/2010	60 IV (30 × 2)	None
		12 IA (6 × 2)	

11/29/1950	7/26/2010	60 IV (20 × 3)	None

		6 IA (6 × 1)	

7/3/1956	11/3/2010	60 IV (24 × 1, 18 × 2)	None

6/1/1942	9/6/2010	90 IV (30 × 3)	None

5/3/1940	4/26/2010	80 IV (40 × 2)	None
		32 IA (16 × 2)	

3/20/1941	8/11/2008	54 IV (27 × 2)	None

3/25/1942	5/5/2008	108 IV (36 × 3)	None

3/5/1963	2/26/2008	99 IV (33 × 3)	None

1/15/1966	8/28/2008	90 IV (45 × 2)	None

2/9/1952	9/30/2009	48 (30 × 1, 18 × 1)	None

* IV = intravenous, IA = intra-articular

## Conclusion

These data suggest the safety and feasibility of administering adipose SVF intravenously. The uses of adipose stem cells have been reported in conditions as diverse as from hearing loss [152], to heart failure [153]. Given the anti-inflammatory, differentiation ability, and trophic factor production by SVF, we are hopeful that these safety data will support ongoing investigation into this novel and easy to access cell population.

## Competing interests

NHR and JPR are shareholders of Medistem Panama and Medistem Inc. None of the other authors have any competing interests.

## Authors' contributions

JPR, MPM, SH, MM, KLM, BM, RJH, CC, RBT, AMM, and NHR performed literature review and wrote the manuscript. SH collected and analyzed patient charts. JPR reported on the clinical cases. NHR, conceived the study and rationale for use of SVF in autoimmunity. All authors read and approved the final manuscript.
